# Transmission of Stability Information through the N-domain of Tropomyosin Is Interrupted by a Stabilizing Mutation (A109L) in the Hydrophobic Core of the Stability Control Region (Residues 97–118)[Fn FN1]

**DOI:** 10.1074/jbc.M113.507236

**Published:** 2013-12-20

**Authors:** J. Paul Kirwan, Robert S. Hodges

**Affiliations:** From the Program in Structural Biology and Biophysics, Department of Biochemistry and Molecular Genetics, School of Medicine, University of Colorado Denver, Aurora, Colorado 80045

**Keywords:** Circular Dichroism (CD), Contractile Protein, Protein Stability, Recombinant Protein Expression, Tropomyosin, Stability Control Region, Tropomyosin N-domain 1–131, Differential Scanning Calorimetry (DSC), Stability Signal Transmission, Two-stranded Coiled-coils

## Abstract

Tropomyosin (Tm) is an actin-binding, thin filament, two-stranded α-helical coiled-coil critical for muscle contraction and cytoskeletal function. We made the first identification of a stability control region (SCR), residues 97–118, in the Tm sequence that controls overall protein stability but is not required for folding. We also showed that the individual α-helical strands of the coiled-coil are stabilized by Leu-110, whereas the hydrophobic core is destabilized in the SCR by Ala residues at three consecutive *d* positions. Our hypothesis is that the stabilization of the individual α-helices provides an optimum stability and allows functionally beneficial dynamic motion between the α-helices that is critical for the transmission of stabilizing information along the coiled-coil from the SCR. We prepared three recombinant (rat) Tm(1–131) proteins, including the wild type sequence, a destabilizing mutation L110A, and a stabilizing mutation A109L. These proteins were evaluated by circular dichroism (CD) and differential scanning calorimetry. The single mutation L110A destabilizes the entire Tm(1–131) molecule, showing that the effect of this mutation is transmitted 165 Å along the coiled-coil in the N-terminal direction. The single mutation A109L prevents the SCR from transmitting stabilizing information and separates the coiled-coil into two domains, one that is ∼9 °C more stable than wild type and one that is ∼16 °C less stable. We know of no other example of the substitution of a stabilizing Leu residue in a coiled-coil hydrophobic core position *d* that causes this dramatic effect. We demonstrate the importance of the SCR in controlling and transmitting the stability signal along this rodlike molecule.

## Teaser

**Background:** A stability control region (SCR) (residues 97–118) is conserved in vertebrate tropomyosins and controls overall stability.

**Results:** An A109L mutation interrupts the transmission of stability information along the coiled-coil, inducing two domains.

**Conclusion:** The optimum stability provided by the SCR is critical for transmission of the stability signal.

**Significance:** Learning how stability information is transmitted along tropomyosin is crucial to understanding function and signaling.

## Introduction

More than 65 years after its initial discovery (1, 2), the actin-binding, coiled-coil protein tropomyosin (Tm)^2^ is a fascinating biological molecule whose structural, stability, and functional properties remain incompletely understood. Tm is most widely known for its role in muscle contraction; its calcium-dependent cooperation with the troponin complex in regulating the interaction of myosin and actin required to generate the power stroke within the sarcomeres of muscle cells (3–6). However, Tm is expressed in all eukaryotic cell types in more than 40 isoforms that are widely distributed within individual cells (7, 8). Consequently, Tm plays a critical role in numerous biologically relevant processes, such as the complex regulation of actin filaments in the cytoskeleton at the leading edge of cells (9, 10), and is associated with several diseases, including cardiomyopathy (11) and cancer (12). Recent reviews document an enormous field of study on Tm function (13–17).

Tropomysoin is a two-stranded, parallel, homodimeric α-helical coiled-coil protein from N terminus to C terminus. The contractile form of the protein is 284 amino acids in length (8) and extends more than 400 Å (18). Tm was the first coiled-coil to have its amino acid sequence determined, which led to the identification of a 3-4 or 4-3 hydrophobic repeating pattern N*XX*N*XXX*N*XX*N*XXX*N …, where N is a nonpolar residue (19, 20). Each sequence of seven amino acid residues in this pattern is known as a heptad and denoted (***abcdefg***)*_n_* (21). Positions ***a*** and ***d*** constitute the hydrophobic core and are typically occupied by non-polar residues that pack like “knobs into holes” (22–24), whereas the ***b***, ***c***, ***e***, ***f***, and ***g*** positions are frequently occupied by polar or charged residues (19, 25–28) with side chains exposed to the surrounding aqueous solvent (23). Coiled-coil structure is adaptable, allowing variation in chain length, parallel and antiparallel orientation, oligomerization states of 2–7 helices, and homomeric or heteromeric oligomerization specificity (29–37). Consequently, the coiled-coil is a frequently occurring motif in biology with considerable structural and functional diversity (36, 38). Tropomyosin's “simple” coiled-coil structure has made it a model coiled-coil for studying the relationships between protein sequence, stability, folding, and function (25) and has stimulated many complementary studies in a variety of coiled-coil systems (36, 39). These approaches are among the most established experimental strategies (25, 28, 36, 40–42) for investigations with broad biological relevance (43).

For many years, our laboratory has investigated tropomyosin and coiled-coil sequence and stability in order to gain insights into folding and function (25, 44–47). Our work has established rules for coiled-coil sequence features. For example, coiled-coil hydrophobic core stability increases with increasing hydrophobicity of the ***a*** and ***d*** residue side chains (32, 48). However, the presence of less stable residues in the ***a*** and ***d*** positions is believed to enable coiled-coil flexibility that is crucial for biological function (25, 32, 34, 49–52). The Tm sequence exhibits alternating regions of hydrophobic core stability (51, 53), where stabilizing clusters > intervening regions > destabilizing clusters (36, 53–55). Stabilizing clusters are regions where non-polar residues occupy three or more consecutive ***a*** and ***d*** positions; destabilizing clusters are regions where polar, charged, or bulky residues occupy three or more consecutive ***a*** and ***d*** positions, and all other sequences comprise intervening regions (46, 47). Residues at outer positions of the coiled-coil (***b***, ***c***, ***e***, ***f***, and ***g***) also contribute to stability through α-helical propensity (54, 56–61), the formation of salt bridge interactions (intrachain, *i* to *i* + *3*, or *i* to *i* + *4* and/or interchain, *i* to *i*′ + *5*) (23, 62–71), trigger sites (72–74), electrostatic clusters (75, 76), and novel hydrophobic interactions from residues at positions ***e*** and ***g*** (70, 76), with the latter two being central to the theme of this study. When mixed and matched, these sequence features enable the fine tuning of stability for different Tm subdomains for optimum function (77–79).

**FIGURE 1.**
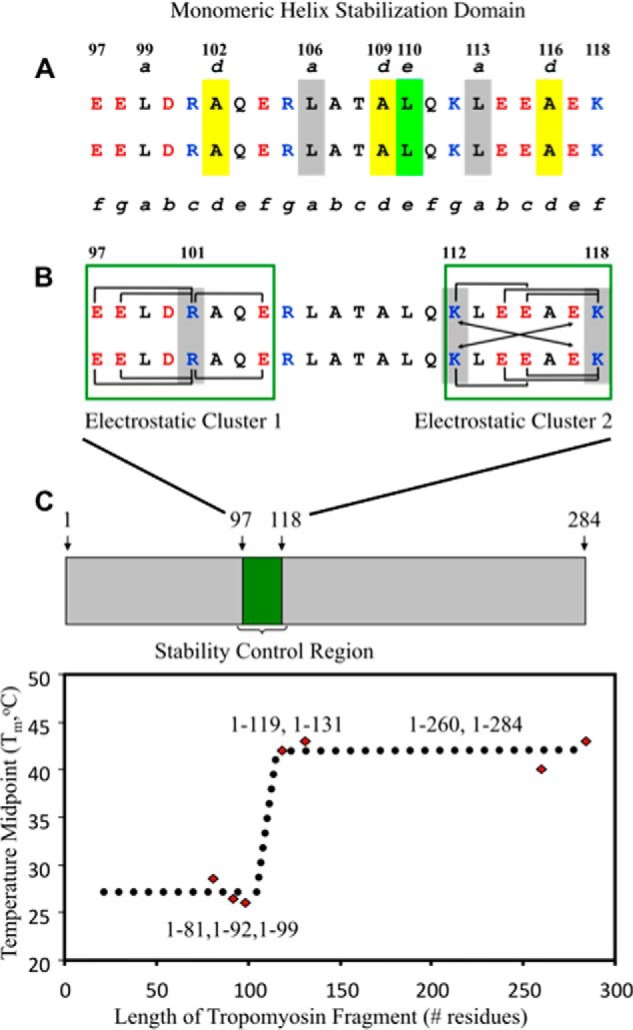
**The stability control region of tropomyosin and its critical interactions.** The Tm sequence 97–118 includes all of the electrostatic and hydrophobic interactions that comprise the stability control region in α-tropomyosin (78). Shown in *A* is the monomeric helix stabilization domain, which consists of *yellow-shaded* Ala residues that occupy three consecutive ***d*** positions (102, 109, 116) in this region and Ala-109***d*** to Leu-110***e*** (shaded in *yellow* and *light green*, respectively) that promote a novel packing arrangement between Leu-106***a*** (*gray*), Leu-110***e*** (*light green*), and Leu-113***a*** (*gray*) (78) within the monomeric helix stabilization domain. In *B*, *green boxes* outline electrostatic clusters 1 and 2, with a large number of intrachain and interchain ionic attractions. The *brackets* denote *i* to *i* + 3 and *i* to *i* + 4 intrachain electrostatic attractions. The *arrows* denote *i* to *i*′ + *5* interchain electrostatic attractions (***g–e*′** and *g*′–***e***). Arg-101***c***, Lys-112***g***, and Lys-118***f*** are critical to the electrostatic clusters and are *shaded* in *gray*. The stability control region was identified from circular dichroism temperature unfolding experiments of tropomyosin C-terminal deletion fragments that showed a 15 °C increase in the *T_M_* (temperature midpoint) value of fragment 1–119 compared with fragment 1–99 (*C* shows the plot of *T_M_* values *versus* tropomyosin fragment length) (77).

**FIGURE 2.**
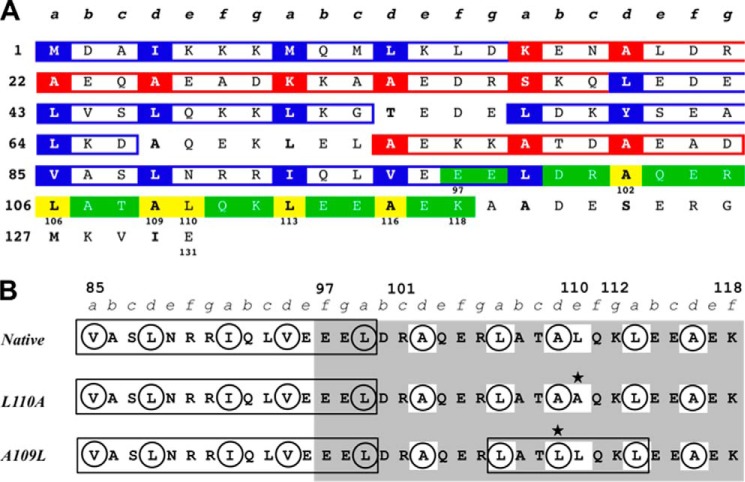
**Tropomyosin amino acid sequence regions 1–131 and 85–119.** The heptad repeat is *labeled **a–g***. *A*, residues in hydrophobic core positions ***a*** and ***d*** are in *boldface type* with stabilizing residues *shaded* in *blue* (Leu, Ile, Val, Met, Phe, and Tyr) and destabilizing residues *shaded* in *red* (all other residues excluding Pro, which is not found in coiled-coils). Three or more stabilizing residues in consecutive hydrophobic core positions (*boxed* in *blue*) constitute a stabilizing cluster. Three or more destabilizing residues in consecutive hydrophobic core positions (*boxed* in *red*) constitute a destabilizing cluster (53). A stabilizing cluster extends into the stability control region *shaded* in *green*. The stability control region includes a destabilized hydrophobic core of three consecutive Ala residues at position ***d*** (Ala-102, Ala-109, and Ala-116) that create a favorable packing for Leu-106***a***, Leu-110***e***, and Leu-116***a*** along the individual α-helices of the coiled-coil (77). These residues constitute a monomeric helix stabilization domain within the stability control region and are *shaded* in *yellow. B*, targeted mutations alter hydrophobic clusters in the stability control region (residues 97–118). Sequence positions of interest are *labeled* with *numbers*. Hydrophobic core residues in positions ***a*** and ***d*** are *circled*, and hydrophobic clusters are *boxed* in *black*. The stability control region is *shaded* in *gray*, and the mutations investigated are *marked* with a *star*. The L110A and A109L mutations both disrupt a monomeric helix stabilization domain (residues *boxed* in *white*) within the stability control region, either by eliminating the interactions between Leu-106***a***, Leu-110***e***, and Leu-113***a*** along each helix (L110A) or by altering their interaction through the addition of a hydrophobic a cluster consisting of Leu-106***a***, Leu-109***d***, and Leu-113***a*** in the hydrophobic core between the helices (A109L).

Recently, we made the first ever identification of a 22-residue stability control region (SCR) (residues 97–118) in Tm (75). The stability control region is a new protein sequence element that provides full-length Tm stability (Fig. 1). Our comparison of the thermal stabilities of a series of N-terminal Tm fragments showed that those lacking the SCR (residues 1–81, 1–92, and 1–99) were able to fold into two-stranded α-helical coiled-coils but were significantly less stable (*T_M_* between 26 and 28.5 °C) than fragments containing the SCR (residues 1–119, 1–131, 1–260, and 1–284). The SCR containing fragments exhibited large increases in thermal midpoint values (*T_M_* 40–43 °C), including a change in *T_M_* (Δ*T_M_*) of +16–18 °C between fragments 1–99 and 1–119 (75). However, the SCR contains an intervening region that includes three Ala residues at consecutive ***d*** positions (102***d***, 109***d***, and 116***d***), each of which destabilizes the coiled-coil by 3.8 kcal/mol relative to a Leu residue (47). This counterintuitive observation challenged us to characterize the critical interactions in the SCR. Using a synthetic peptide approach (76), we revealed three stability control sites within the SCR: two electrostatic clusters, EELDRAQE (residues 97–104) and KLEEAEK (residues 112–118), and a third site, RLATALQ (residues 105–111), that is central to a unique hydrophobic packing arrangement in the SCR. The analog sequences R101A and K112A/K118A disrupted an abundance of potential stabilizing intrachain and interchain salt bridge interactions and resulted in a 7 °C loss in *T_M_* value for each analog relative to the wild type peptide sequence. However, an L110A substitution remarkably resulted in a 2.7 kcal/mol decrease in stability in the monomeric α-helices of the coiled-coil (76) (Fig. 1). The loss of a Leu residue at position 110***e*** disrupts favorable hydrophobic interactions between Leu-106***a***, Leu-110***e***, and Leu-113***a*** along the individual strands of the coiled-coil. These hydrophobic interactions collectively constitute a monomeric helix stabilization domain within the SCR (Figs. 1 and 2*A*) that is able to exist because of the minimized hydrophobic surface areas of the destabilizing Ala residues at 102***d***, 109***d***, and 116***d*** (76). We contend that the SCR maintains an optimum stability that must be critical for Tm function.

Here we investigated how the effects of critical mutations in the SCR affect optimum stability within a large N-terminal fragment of Tm. The stability control region (residues 97–118) is located within the N-terminal half of the Tm molecule (residues 1–142). Previous studies document that the N-terminal half of Tm is more stable than the C-terminal half (80–82). Furthermore, neither N-terminal nor C-terminal fragments lacking the SCR exhibit full-length Tm stability (75, 83). We chose to investigate critical SCR mutations in Tm(1–131) because of our previous experience studying this fragment (75), because of its similarity to another previously studied N-terminal fragment, Tm(1–133) (81), and because we wanted to focus on how changes in the SCR affect the N terminus of Tm. We prepared three recombinant constructs encoding the rat Tm fragment 1–131, including the wild type sequence, an L110A mutation (76), and an A109L mutation. Each of these constructs included an Ala-Ser N-terminal dipeptide, (ASTm(1–131); see “Experimental Procedures”). The L110A substitution was expected to destabilize Tm(1–131) because it dramatically reduced stability (−2.7 kcal/mol) in synthetic peptides of the stability control region (76). In contrast, the A109L mutation was expected to stabilize Tm(1–131) by introducing a canonical Leu at position ***d*** and adding a three-residue stabilizing cluster within the stability control region (Fig. 2). The three proteins were purified and analyzed by circular dichroism (CD) and differential scanning calorimetry (DSC). The L110A mutation did destabilize Tm(1–131) relative to wild type, but the single mutation A109L simultaneously stabilized and destabilized different regions of Tm(1–131), which was an unexpected result that has never been observed before. These results suggest a fascinating phenomenon, that the SCR transmits its control throughout the N-terminal domain of Tm and that single mutations in the stability control region (residues 97–118) can disrupt its effect. We propose that the SCR accomplishes this through novel interactions, such as the stabilization of the monomeric α-helices, which optimize stability to allow dynamic motion that is essential for the transmission of stabilizing information along the Tm coiled-coil.

## EXPERIMENTAL PROCEDURES

### 

#### 

##### Preparation of Rat α-Tropomyosin Tm(1–131) DNA Constructs

We used a pET11d (Novagen, EMD Millipore, Billerica, MA) plasmid construct (courtesy of Sarah E. Hitchcock-DeGregori, Robert Wood Johnson Medical School, Brunswick, NJ) containing the rat α-tropomyosin DNA sequence with an Ala-Ser dipeptide on the N terminus as described elsewhere (84). The rat α-Tm sequence differs from human α-Tm only at one position (R220K). The construct was transformed into competent *Escherichia coli* DH5α and BL21 (DE3) cells. Multiple 5-ml cultures of these cells were grown in LB with 100 μg/ml ampicillin for Miniprep plasmid DNA preparations (Qiagen, Hilden, Germany) to make a stock of Tm/pET11d plasmid. The rat Tm gene was verified by DNA sequencing. The Tm/pET11d construct (encoding full-length Tm, residues 1–284) was used as template DNA for polymerase chain reaction (PCR) site-directed mutagenesis (Stratagene QuikChange XL, Agilent, Santa Clara, CA) to insert stop codons to create a Tm gene product length of 1–131. The resulting construct was verified by DNA sequencing and then used as template DNA to create two additional constructs whose Tm genes encoded one of two mutations, A109L and L110A. Reaction volumes were 25 or 50 μl and included excess DNA template or 1 μl of dimethyl sulfoxide (DMSO) in some cases. Each of the three resulting constructs (Tm(1–131) wild type, A109L, and L110A) were verified by DNA sequencing. These plasmids were transformed into competent *E. coli* DH5α and BL21 (DE3) cells.

##### Bacterial Expression

Cultures (50 ml) of *E. coli* BL21 (DE3) cells containing the rat Tm/pET11d constructs in LB with 100 μg/ml ampicillin were shaken overnight at 37 °C. The overnight cultures were transferred to 1-liter cultures of LB with 100 μg/ml ampicillin and shaken at 37 °C for 4–6 h. The 1-liter cultures were then induced with 0.5 mm isopropyl-β-d-1-thiogalatopyranoside and shaken overnight at room temperature. Cells were harvested by centrifuge in a high capacity rotor at 7,000 × *g*. The supernatant was decanted from the cell pellet, which was either stored overnight at 20 °C for later use or frozen at −80 °C for 1–2 h and then thawed on ice for immediate use.

##### Sonication of Harvested Cells and Extraction of Rat Tm(1–131) Proteins

Frozen cell pellets from 1 liter of expression culture were thawed on ice for 30 min and then resuspended with vortex mixing in 40 ml of aqueous 1% trifluoroacetic acid (TFA) per pellet, transferred to a 50-ml conical tube, and mixed by rocking at room temperature for 30 min. The samples were then sonicated three times for 30 s each at 55% power using a Fisher Sonic Dismembrator model 500 (Thermo Fisher Scientific) with mixing by inversion after each round. The sonicated samples were then mixed by rocking at room temperature for 30 min and centrifuged for 10 min at 10,000 × *g*. The resulting insoluble pellet was stored at −20 °C. The supernatant (soluble fraction) was decanted to a separate container and lyophilized. A small amount of the soluble fraction was removed for analytical reversed-phase HPLC and LC-MS analyses. The resulting lyophilized material was weighed and documented as crude sample. The amount of lyophilized soluble crude material obtained from a 1-liter expression was 200–350 mg.

##### One-step RP-HPLC Purification and Mass Verification

Crude Tm proteins were purified by a one-step RP-HPLC method similar to that described by our laboratory (85). Lyophilized crude samples were weighed, reconstituted in aqueous 0.2% TFA (mobile phase A) at ∼2 mg/ml, passed through 0.2-μm (PVDF) filters (EMD Millipore, Billerica, MA), and injected onto an Agilent 1100 series or Beckman Gold preparative high performance liquid chromatograph (Beckman Coulter, Brea, CA) with a Zorbax 300SB-C8 250 × 9.6-mm inner diameter semiprep reversed-phased HPLC column (Agilent). The crude samples were then purified using two mobile phases (A, aqueous 0.2% TFA; B, acetonitrile, 0.18% TFA) and a preparative gradient program of 0% B to 25% B at 2% B/min, followed by 25% B to 45% B at 0.1% B/min, and a concluding column wash step of 90% B for 10 min. Fractions were collected every minute (2 ml). Pure fractions were identified by analytical HPLC on the Agilent 1100 system with a narrow bore Zorbax-300SB 150 × 2.1-mm inner diameter reversed-phase column (Agilent) with a gradient of 1%B/minute and masses were confirmed by liquid chromatography-mass spectrometry (LC-MS) using an Agilent 1100 series mass selective detector ion trap system (LC/MSD Trap SL).

##### Rat Tm(1–131) Protein Sample Aliquot Preparation

Purified HPLC fractions were pooled, lyophilized, and reconstituted at 1 mg/ml by weight. 30 μl (∼30 μg) were removed for amino acid analysis to determine protein concentration. Individual aliquots of 250 or 500 μg of pure protein were ready for reconstitution with high reproducibility.

##### Amino Acid Analysis

Triplicate aliquots of ∼30 μg of purified Tm(1–131) proteins were mixed with 300 μl of 6 m HCl, 1% phenol in glass vials and heated to 100 °C for 48 h for complete hydrolysis of the proteins. The hydrolysates were dried under vacuum and reconstituted for derivatization and analysis on an 1100 series Agilent high performance liquid chromatograph equipped with a Waters 3.9 × 150-mm inner diameter column using the Waters (Milford, MA) AccQ-Tag Method^TM^, as originally described elsewhere (86).

##### Sample Preparation for CD Spectroscopy and DSC

Sample aliquots of 250 μg to 1 mg were reconstituted in 500 μl to 1 ml of benign buffer (100 mm KCl, 50 mm PO_4_, pH 7), injected into a 500-μl to 3-ml Slide-a-Lyzer dialysis cassette (Thermo Fisher Scientific) with a 3,500 molecular weight cut-off, dialyzed overnight, and then recovered from the dialysis cassette for CD and DSC analysis.

##### CD Spectroscopy and Data Analysis

The Tm(1–131) protein samples analyzed by CD and DSC were prepared from the same dialyzed aliquot. CD spectroscopy was performed on a Jasco J-815 spectropolarimeter (Jasco, Inc., Easton, MD). A Lauda model RMS circulating water bath (LAUDA-Brinkman, Lauda-Brinkman, Lauda-Konigshofen, Germany) was used for thermal uniformity for the PFD-452S Peltier temperature controller that maintains the temperature control of the optical cell. CD absorbance is expressed as molar ellipticity, [θ] (degrees·cm^2^·dmol^−1^) and calculated from the equation,

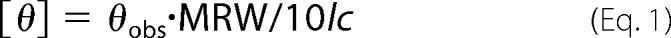
 where θ_obs_ is the observed ellipticity in millidegrees, MRW is the mean residue weight of the protein (molecular weight/number of residues), *l* is the optical path length of the cell in centimeters, and *c* is the concentration in mg/ml. Samples were prepared as described above and then serially diluted to 0.2–0.5 mg/ml for CD analysis in a 0.5-mm path length cell. Variable wavelength measurements (spectrum scans) of protein solutions were scanned at 5 °C from 195 to 250 nm, with data points collected every 0.2 nm and a scan rate 50 nm/min. The average of six scans was recorded for each experiment. A spectrum scan was performed at 5 °C before each protein was melted, after each protein was melted once the temperature had returned to 5 °C, and again after 15 min at 5 °C. The average of the three experiments was reported for each protein in Table 1. Variable temperature measurements (thermal denaturation or melt) of proteins in benign buffer were scanned at 222 nm from 5 to 75 °C in 0.2 °C increments with a scan rate of 60 °C/h. Thermal denaturation profiles were displayed in molar ellipticity at 222 nm or fraction folded (relative CD, normalized between 0 and 1) *versus* temperature, according to the equation,

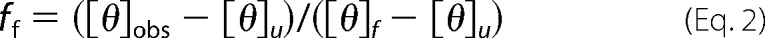
 where [θ]_obs_ is the observed molar ellipticity at a given temperature, [θ]*_u_* is the molar ellipticity of the fully unfolded species, and [θ]*_f_* is the molar ellipticity of the fully folded species. By convention, the temperature midpoint (*T_M_*) corresponds to an *f_f_* value of 0.5, or 50% folded peptide, and was determined from fitting the curves of the thermal denaturation profiles to the Gibbs-Helmholtz equation to determine the T*_M_*,

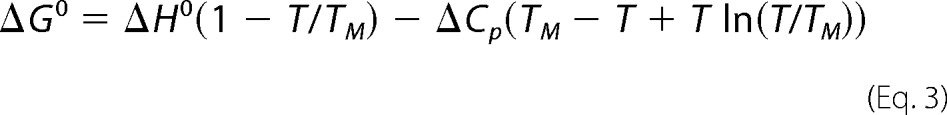
 the van't Hoff enthalpy of unfolding (Δ*H_u_*), and the apparent free energy of unfolding (Δ*G_u_*_,app_), as described elsewhere (87) and in the supplemental Methods. Errors were determined in CD measured values from the S.D. of the three scans or melts and were propagated to determine the errors of calculated values.

##### DSC and Data Analysis of Rat Tm(1–131) Proteins

Dialyzed aliquots of Tm(1–131) were analyzed by DSC using a Microcal VP-DSC (General Electric, Fairfield, CT) to measure the excess heat capacity of proteins unfolding as a function of temperature and directly calculate the calorimetric enthalpy of thermal unfolding (Δ*H*_Cal_) of the Tm(1–131) proteins according to Equation 4.

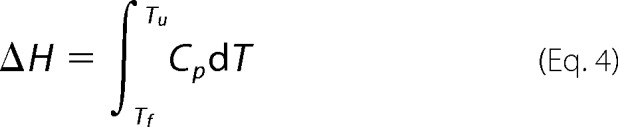
 Samples were prepared as described above and serially diluted to 0.5–0.75 mg/ml (20–50 μm) for DSC analysis measuring excess *C_p_* over the temperature range of 5 °C to 75 °C and a scan rate of 60 °C/h. Buffer baseline scans were established prior to analysis of each protein by performing three or more consecutive heating and cooling scans with overnight dialysis equilibrated buffer in both the sample and reference cells until the scans deviated less than 0.00005 kcal/°C. Buffer was then removed from the sample cell, and sample was loaded between 25 and 15 °C during cooling with a system pressure of ≥30 p.s.i. Samples were scanned through three consecutive heating and cooling cycles. Data were analyzed using the Origin version 6.0 software packaged with the Microcal VP-DSC. Sample scans were buffer-subtracted, concentration-normalized, corrected with the *progress baseline* option, and fitted by nonlinear least squares analysis using the *non-two-state* model option. The DSC curves for all three proteins were best fit with three transitions (lowest χ-squared values by a factor of 1000). The resulting fitted excess heat capacity curves yielded the melting temperature (*T_M_*), Δ*H*_cal_, and Δ*H*_vH_. The *T_M_* and Δ*H* values were averaged from these scans for each protein and reported in Table 2. Errors were determined from the S.D. of three scans and were propagated to determine the errors of calculated values.

## RESULTS

**FIGURE 3.**
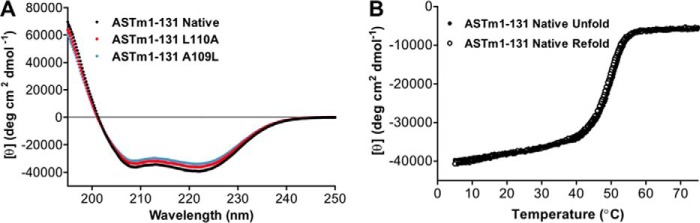
**Folding and reversibility of Tm(1–131) wild type and mutant proteins using CD spectroscopy.** *A*, CD spectrum scans of Tm(1–131) wild type (*black*), L110A (*red*), and A109L (*light blue*) measured immediately after thermal denaturation to 75 °C and cooling back to 5 °C. These scans indicate helical structure with very little difference in helical content between wild type and mutants. *B*, overlay of thermal denaturation or unfolding (*dark circles*) and refolding (*open circles*) profiles for wild type Tm(1–131) using CD. The profiles are shown overlapping, indicating equilibrium unfolding (reversibility of folding) in the temperature range of 5–75 °C. Tm(1–131) L110A and A109L both exhibited similar overlapping unfolding and refolding profiles. *ASTm(1–131)* indicates the presence of an N-terminal Ala-Ser dipeptide in these Tm(1–131) sequences. All profiles were measured with a temperature change of 1 °C/min.

**FIGURE 4.**
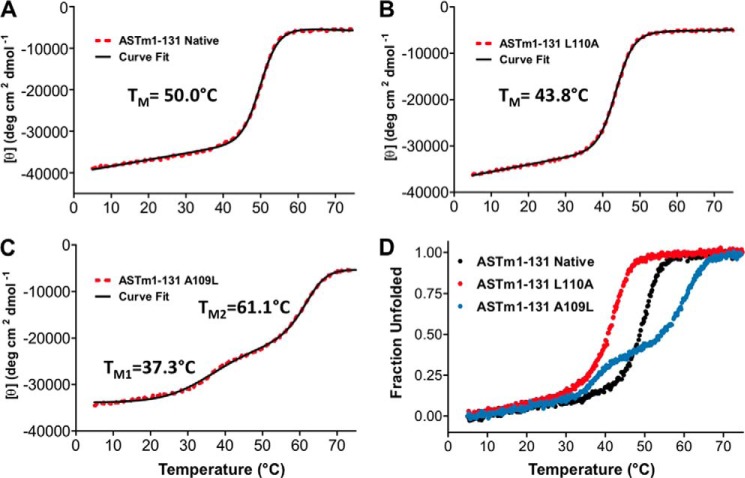
**Thermal denaturation profiles of wild type and mutant Tm(1–131) proteins using CD spectroscopy.** Shown are the Tm(1–131) protein unfolding profiles (*red dots*) (*A*, wild type; *B*, L110A; *C*, A109L) with their associated nonlinear least-square fits (*black lines*) (89). *D*, the overlaid profiles for wild type (*black*), L110A (*red*), and A109L (*blue*) are shown in fraction folded. *A* and *B* show apparent single-transition profiles with characteristic pretransition coiled-coil baselines. *C* shows two distinct transitions induced by the single mutation A109L, which simultaneously increases and decreases stability in different regions of the molecule. *ASTm(1–131)* indicates the presence of an N-terminal Ala-Ser dipeptide in these Tm(1–131) sequences. All profiles were measured with a temperature change of 1 °C/min.

Overlaid CD Spectra (195–250 nm) of Tm(1–131) wild type, L110A, and A109L indicated that each protein was fully folded, with [θ]_222_ (degrees·cm^2^·dmol^−1^) values of 39,143 (wild type), 36,412 (L110A), and 34,587 (A109L) in benign conditions (100 mm KCl, 50 mm PO_4_, pH 7) (Fig. 3*A* and Table 1). The [θ]_222_/[θ]_208_ ratios for these proteins were greater than 1, indicating that all three proteins were folded as coiled-coils (88). However, it was interesting that although A109L was expected to be the most stable mutant, it showed the least helical content of the three proteins (89). Initial thermal unfolding CD experiments (5–75 °C, 1 °C/min) were immediately cooled at the same rate (75–5 °C, 1 °C/min) for comparison of the unfolding and refolding profiles of each protein. Fig. 3*B* shows that the unfolding and refolding profiles of Tm(1–131) wild type overlay, indicating that the sample was in thermodynamic equilibrium. The unfolding and refolding profiles of Tm(1–131) L110A and A109L overlapped similarly (data not shown) and indicated that the unfolding of all of our samples was reversible. We proceeded to compare the thermal stabilities of the Tm(1–131) proteins evaluated by CD and observed novel results in which the L110A and A109L mutations completely altered the stability of this N-terminal domain in very different ways. Fig. 4 shows their melting profiles and associated non-linear least squares fit according to the Gibbs-Helmholtz equation (Equation 3). Relative to wild type, L110A destabilized the Tm(1–131) protein by more than 6 °C (Fig. 4, *A* and *B*, and Table 1). However, A109L simultaneously stabilized 55% of the molecule by more than 11 °C and destabilized 45% of the molecule by more than 12 °C, relative to wild type, based on the fractions of total ellipticity associated with each of two apparent transitions (Fig. 4, *A* and *C*, and Table 1). We had expected A109L to increase the stability of the entire protein, but instead Tm(1–131) was divided into two domains of stability, as is clearly shown in Fig. 4*D*.

**TABLE 1:** **Biophysical data for Tm(1–131) proteins analyzed by CD spectroscopy**

Protein	[θ]_222_*^a^*	[θ]_222_/[θ]_208_*^b^*	% Helix*^c^*	T*_M_**^d^*	ΔT*_M_**^e^*
			%		
Native	39,143 ± 935	1.09 ± 0.04	107 ± 2.6	50.0 ± 0.0	
L110A	36,412 ± 549	1.08 ± 0.02	99 ± 1.5	43.8 ± 0.2	6.2 ± 0.2
A109L-1*^f^*	34,587 ± 751	1.08 ± 0.04	94 ± 2.0	37.3 ± 0.2	−12.7 ± 0.2
A109L-2*^f^*				61.1 ± 0.3	+11.1 ± 0.3

*^a^* Mean residue ellipticity from CD spectra at 222 nm in benign buffer (100 mm KCl, 50 mm PO_4_, pH 7) at 5 °C. Protein concentration ranged from 29 to 34 μm monomer. Ellipticity values shown are the average of triplicate experiments with error of ≤2.5%.

*^b^* Ratio of mean residue ellipticity at 222 and 208 nm, benign buffer.

*^c^* Percentage helix is calculated from [θ]_H_*^n^* = [θ]_H_^∞^ (1 − *k*/*n*) where [θ]_H_^∞^ = −37,400 degrees cm^2^ dmol^−1^ for a helix of infinite length, *n* is the number of residues in the helix, and *k* is a wavelength-dependent constant (2.5 at 222 nm). For a 133-residue protein, the theoretical value for 100% helix is −36,697 degrees cm^2^ dmol^−1^ (91).

*^d^ T_M_* is the temperature at which 50% of the protein is unfolded.

*^e^* Change in *T_M_* relative to the wild type protein.

*^f^* A109L-1 and A109L-2 refer to A109L domain 1 and A109L domain 2, respectively.

**FIGURE 5.**
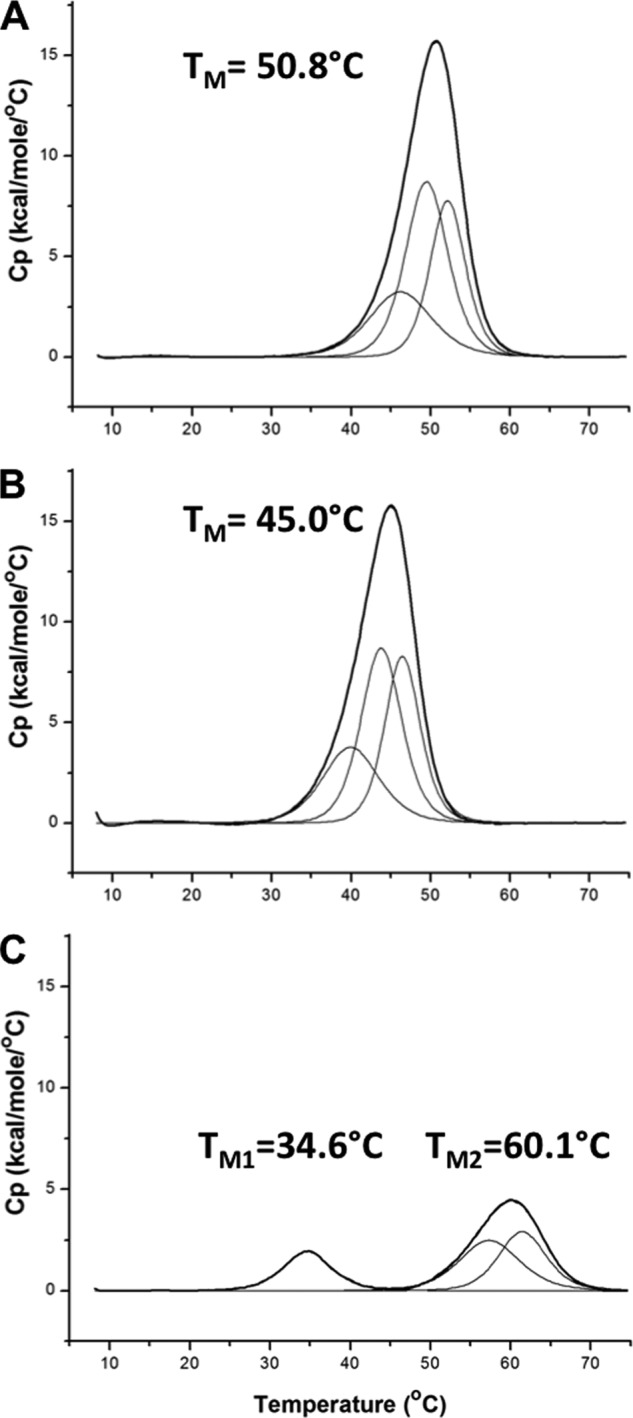
**Thermal denaturation profiles of wild type and mutant Tm(1–131) proteins using DSC.** Profiles of the excess heat capacity (*C_p_*) of Tm(1–131) proteins (*A*, wild type; *B*, L110A; *C*, A109L) are shown (*thick black lines*). The scans were buffer-subtracted, normalized for concentration, and baseline-corrected. In each case, the excess heat capacity profiles were best fit by deconvolution into three components (*thin black lines*) that sum to fit the observed profiles exactly. *A* and *B* show apparent single-transition profiles, with *T_M_* values of 50.8 °C (wild type) and 45.0 °C (L110A), respectively, with each profile composed of three components. *C* shows that the A109L mutation induces two apparent transitions in the excess heat capacity profile, where domain 1 (*T_M1_* of 34.6 °C) consists of a single component and domain 2 (*T_M2_* of 60.1 °C) consists of two components. All profiles were measured with a temperature change of 1 °C/min. Data analysis was performed with Origin version 7 software included with the Microcal VP-DSC instrument.

Our CD results were confirmed and enhanced by the analysis of these proteins by DSC. To ensure the best comparison possible, the DSC samples were taken from the same sample aliquot used to prepare the CD samples, and both experiments were performed at the same time. As observed in the CD, L110A was destabilized relative to wild type by 6 °C (Fig. 5, *A* and *B*, and Table 2). A109L was divided into two domains, one domain that was stabilized by more than 9 °C and another domain that was destabilized by more than 16 °C relative to wild type (Fig. 5, *A* and *C*, and Table 2). Deconvolution of the excess heat capacity curves revealed that all three proteins unfold in three transitions (Fig. 5, *A–C*), which is consistent with previous results in full-length Tm (77, 90, 91). The additional detail from DSC showed that the A109L mutation caused the destabilization of transition 1, which resulted in the formation of domain 1, but stabilized transitions 2 and 3 to form domain 2. The A109L mutation appeared to decouple the energetic relationship between domains 1 and 2. The observation of multiple transitions in Tm(1–131) is consistent with previous investigations of wild type Tm(1–284) (77, 91, 92). Unfolding of Tm(1–284) has also revealed total enthalpy values of ∼300 kcal/mol (90, 91, 93), suggesting a total enthalpy value for wild type Tm(1–131) of ∼140 kcal mol^−1^. Our measured total enthalpy value for wild type Tm(1–131) was 142.5 kcal mol^−1^, which is consistent with previous results. However, there was little difference between wild type and L110A (144.5 kcal mol^−1^). These results suggest that the wild type Leu at 110***e*** contributes an entropic benefit to the stability control region, although total entropy values are similar also for wild type (0.441 kcal K^−1^ mol^−1^) and L110A (0.456 kcal K^−1^ mol^−1^). The total enthalpy and entropy values for A109L (66.6 kcal mol^−1^ and 0.441 kcal K^−1^ mol^−1^) were less than half of the values of wild type and L110A, which is consistent with a disruption in approximately half of the structure. We know of no other example of the substitution of a canonical, stabilizing Leu residue in a coiled-coil hydrophobic core position ***d*** that causes the dramatic destabilization observed in Tm(1–131) A109L. The unexpected effect of A109L shows the crucial balance in stability provided by the stability control region and how little it tolerates sequence modifications that cause a deviation from optimal stability.

**TABLE 2:** **Biophysical data for Tm(1–131) proteins analyzed by differential scanning calorimetry**

Protein transition	*T_t_**^a^*	kcal mol^−1^ ΔH_cal,t*^b^*_	kcal mol^−1^ ΔH_vH,t*^c^*_	kcal K^−1^ mol^−1^ Δ*S_t_**^d^*	Domain*^e^*	*T_M_**^f^*	Δ*T_M_**^g^*	kcal mol^−1^ ΣΔ*H*_cal*^h^*_	kcal *K*^−1^ mol^−1^ ΣΔ*S**^i^*	kcal mol^−1^ Δ*G*_*u*,25 °C*^j^*_	kcal mol^−1^ ΔΔ*G*_*u*,25 °C*^k^*_
	°*C*					°*C*	°*C*				
Native-1	46.4 ± 0.2	35.6 ± 1.9	78.5 ± 1.9	0.111 ± 0.006	1						
Native-2	49.6 ± 0.1	62.0 ± 1.5	117.3 ± 1.1	0.192 ± 0.005	1	50.8 ± 0.1	–	142.5 ± 2.8	0.441 ± 0.009	10.9 ± 0.3	–
Native-3	52.2 ± 0.0	44.9 ± 1.5	142.1 ± 1.3	0.138 ± 0.005	1						
L110A-1	40.3 ± 0.1	39.4 ± 0.7	81.1 ± 0.3	0.126 ± 0.002	1						
L110A-2	44.1 ± 0.0	63.5 ± 1.4	116.8 ± 0.8	0.200 ± 0.004	1	45.0 ± 0.0	−5.2 ± 0.1	144.5 ± 2.2	0.456 ± 0.007	8.56 ± 0.2	2.31 ± 0.4
L110A-3	46.6 ± 0.0	41.6 ± 1.6	140.4 ± 1.1	0.130 ± 0.005	1						
A109L-1	34.7 ± 0.2	17.4 ± 4.1	92.3 ± 13.2	0.057 ± 0.013	1	34.6 ± 0.3	−16.2 ± 0.3				
A109L-2	57.0 ± 0.5	26.5 ± 1.6	81.3 ± 7.5	0.080 ± 0.005	2	60.1 ± 0.1	+9.3 ± 0.1	67.4 ± 5.3	0.207 ± 0.017	5.67 ± 0.6	5.21 ± 0.7
A109L-3	61.3 ± 0.3	23.5 ± 3.0	114.1 ± 4.6	0.070 ± 0.009	2						

*^a^ T_t_* is the temperature at the midpoint of the transition determined by deconvolution.

*^b^* Calorimetric enthalpy values were determined from a direct fit of the excess heat capacity. A non-two-state model with three transitions provided the best fit (lowest χ^2^ value).

*^c^* The van't Hoff enthalpy values were calculated for each transition in the non-two-state unfolding profile.

*^d^* Each transition can be treated as an individual two-state process, where Δ*S_t_* = Δ*H_t_*/*T_t_*.

*^e^* The wild type and L110A unfolding profiles exhibit one apparent domain comprising three transitions. A109L shows two apparent domains, where domain 1 contains transition 1, and domain 2 contains transitions 2 and 3.

*^f^* T*_M_* is the temperature at the midpoint of the apparent unfolding domain.

*^g^* Change in T*_M_* relative to the wild type protein.

*^h^* The total enthalpy of unfolding is the sum of the Δ*H*_cal,*t*_ values for each transition.

*^i^* The total entropy of unfolding is the sum of the Δ*S_t_* values for each transition.

*^j^* The total free energy of unfolding was calculated from the Gibb's equation, Δ*G_u_* = ΣΔ*H* − *T*ΣΔ*S*, at 25 °C.

*^k^* Change in Δ*G_u_* relative to the wild type protein.

## DISCUSSION

We have observed that a single mutation (L110A) in the stability control region completely destabilizes the first 131 residues of Tm. This result shows that the destabilizing effect of L110A in the SCR is transmitted through the entire 1–131 sequence region, corresponding to a distance of ∼165 Å from the substitution site at residue 110 to residue 1. The single mutation A109L causes an even more dramatic effect on stability, simultaneously stabilizing and destabilizing different portions of the Tm(1–131) molecule relative to wild type. We expected the A109L mutation to stabilize the SCR and the entire Tm molecule by adding a Leu to the hydrophobic core position ***d***. However, the excessive stabilization of the SCR by A109L effectively decoupled the SCR from an N-terminal portion of the molecule, resulting in an overall decrease in stability of Tm(1–131) A109L that was completely unexpected. The effects of both the L110A and A109L mutations are unprecedented in a long coiled-coil of 131 amino acids and are dramatically different despite being one residue apart in the sequence. Our results demonstrate the importance of the SCR in controlling stability and transmitting information along the coiled-coil sequence. This indicates that coiled-coil mutations not only alter regional stability (69) but can also affect stability at distal sites.

Our DSC results suggest that a Leu at position 110***e*** (wild type) provides an entropic benefit to the SCR. This is consistent with the favorable entropic benefit associated with hydrophobic core packing in GCN4 (94, 95). Similarly, the packing of Leu-106***a***, Leu-110***e***, and Leu-113***a*** along the monomeric α-helices (*i.e.* monomeric helix stabilization domain) in the stability control region enhances this effect by limiting the exposure of Leu-110***e*** to solvent, which shields the hydrophobic core (70, 76). However, because interchain hydrophobic core interactions (between strands) are minimized, the individual α-helices have greater potential for dynamic movement. Effectively, the entropic benefit gained from L110***e*** (an intact SCR) enables the SCR to efficiently distribute a stabilizing contribution (96) throughout a large region of the molecule that would otherwise be impossible with canonical Leu residues at ***d*** positions in the SCR. The L110A mutation induces the transmission of a destabilizing signal from the SCR that is propagated along the sequence, destabilizing each heptad between the stability control region and the N terminus of Tm. In contrast, the A109L mutation increases stability in the SCR, locking down the hydrophobic core and stabilizing the C terminus. However, the transmission of stability to the N terminus is interrupted by the excessive stabilization of the SCR, resulting in two domains of stability. Because the less stable N-terminal domain of Tm(1–131) A109L receives no transmitted signal, its stability is similar to the Tm fragments 1–81, 1–92, and 1–99, which lack the SCR altogether (75).

The significance of our results is apparent, especially considering that the relationship between protein stability and the cooperativity required for function is still incompletely characterized (43). We envision a stability propagation model in Tm, where the SCR transmits stabilizing information throughout the molecule. Such global communication is of strong general interest and under active investigation (97, 98). For example, molecular dynamics simulations in Tm suggest that clusters of Ala residues in the hydrophobic core (Ala***a***-Ala***d***-Ala***a*** or Ala***d***-Ala***a***-Ala***d***) “relay” a curvature signal in Tm that is delocalized and long range (99, 100). These simulations also showed that stabilized Tm mutants (A74L/A78V/A81L) (77) exhibit reduced curvature and overall straightening that is propagated throughout the molecule to promote efficient Tm-actin interaction in the thin filament (77, 99, 100). Similarly, our concept of the rapid transmission of stability through the SCR provides a compelling explanation for the efficient Tm response during muscle contraction. The Tm-associated troponin subunit, TnT (101, 102), binds to Tm between Cys-190 and the C-terminal/N-terminal overlap of an adjacent Tm molecule in the thin filament (103–106). Consequently, proper transmission of stability information through the SCR could facilitate the relay of Ca^2+^-mediated changes throughout the Tm molecule and the thin filament (55, 107, 108). In this context, a Tm L110A mutant is likely to exhibit an excessively flexible structure with deficient actin-Tm-troponin cooperative switching during muscle contraction (99, 109–112). In contrast, a Tm A109L mutant with dramatically different domains of stability may not bind to actin at all (77).

Evidence exists to support our assertion that the SCR is required for proper Tm function. The SCR is contained in Period 3 (75, 76) of the seven quasiequivalent actin-binding periods in Tm (113) and is conserved in all vertebrate Tm isoforms (113, 114). Loss of Period 3 leads to reduced myosin cycling (109, 110) and reduced contractile force (115–117). In addition, among the 13 known cardiomyopathy mutations in Tm (118, 119), we observe that none occur in the SCR, but they are instead evenly distributed in either “half” (N-terminal or C-terminal) of the Tm sequence. This suggests that mutations in the SCR are embryonic lethal and therefore never observed. Interestingly, the closest cardiomyopathy mutation to the SCR, V95A, is associated with a mild cardiac phenotype (minimal change in thin filament structure) but high morbidity (120). Similarly to the loss of Period 3, Tm V95A results in a destabilized actin-myosin complex, reduced S1-ATPase activity, and delayed myosin cycling (111, 120). Tm V95A changes a five-residue stabilizing cluster (Val-85/Leu-88/Ile-92/Val-95/Leu-99) to three and extends an intervening region (Ala-95/Leu-99/Ala-102/Leu-106/Ala-109/Leu-113/Ala-116) from within the SCR (Fig. 2). We suspect Tm V95A-induced cardiomyopathy to be a manifestation of altered stability in the SCR.

The essence of the observed effects of the Tm(1–131) mutants L110A and A109L clearly indicates the critical requirement for optimum stability in the SCR. Optimum stability allows functionally beneficial dynamic motion that is critical for the transmission or propagation of stabilizing information along the coiled-coil. We predict that an intact SCR with optimum stability is required for proper Tm function.

## Footnotes

2 The abbreviations used are:

Tm: tropomyosin
SCR: stability control region
DSC: differential scanning calorimetry.
